# Classification Framework for Healthy Hairs and Alopecia Areata: A Machine Learning (ML) Approach

**DOI:** 10.1155/2021/1102083

**Published:** 2021-08-14

**Authors:** Choudhary Sobhan Shakeel, Saad Jawaid Khan, Beenish Chaudhry, Syeda Fatima Aijaz, Umer Hassan

**Affiliations:** ^1^Department of Biomedical Engineering, Ziauddin University, Faculty of Engineering, Science, Technology and Management, Karachi, Pakistan; ^2^School of Computing and Informatics, University of Louisiana at Lafayette, USA

## Abstract

Alopecia areata is defined as an autoimmune disorder that results in hair loss. The latest worldwide statistics have exhibited that alopecia areata has a prevalence of 1 in 1000 and has an incidence of 2%. Machine learning techniques have demonstrated potential in different areas of dermatology and may play a significant role in classifying alopecia areata for better prediction and diagnosis. We propose a framework pertaining to the classification of healthy hairs and alopecia areata. We used 200 images of healthy hairs from the Figaro1k dataset and 68 hair images of alopecia areata from the Dermnet dataset to undergo image preprocessing including enhancement and segmentation. This was followed by feature extraction including texture, shape, and color. Two classification techniques, i.e., support vector machine (SVM) and *k*-nearest neighbor (KNN), are then applied to train a machine learning model with 70% of the images. The remaining image set was used for the testing phase. With a 10-fold cross-validation, the reported accuracies of SVM and KNN are 91.4% and 88.9%, respectively. Paired sample *T*-test showed significant differences between the two accuracies with a *p* < 0.001. SVM generated higher accuracy (91.4%) as compared to KNN (88.9%). The findings of our study demonstrate potential for better prediction in the field of dermatology.

## 1. Introduction

The “falling of scalp hairs in sufficient quantity” is defined as hair loss [1]. Alopecia areata is an autoimmune disorder that involves nonscarring hair loss in well-defined patches that can affect the entire scalp region and, ultimately, lead to baldness [2, 3]. The disorder impacts millions of people worldwide [4], especially those with a family history of alopecia areata [5]. It begins when the body's autoimmune system starts to target the hair follicles, disturbing their normal functioning and preventing subsequent hair growth. The outcome is hair loss. Hair loss can be attributed to various causes, and trichoscopies and biopsies are generally necessary to ensure the cause is alopecia areata. However, the limitations of these diagnostic methods are the uncertainty surrounding the number of tests required for adequate diagnosis. Hence, there is a vast scope for researching new techniques pertaining to the classification and diagnosis of alopecia areata [6].

Machine learning (ML) techniques have shown effectiveness in the prediction and classification of various diseases and disorders. Machine learning encapsulates the study of different computer algorithms that exhibit the potential to learn and adapt [7]. Machine learning (ML) algorithms and their advanced versions have been incorporated in various medical disciplines for diagnostic purposes. For instance, machine learning (ML) techniques have exhibited accurate results using magnetic resonance imaging (MRI) and computed tomography (CT) images for the diagnosis of brain tumors, breast cancer, ovarian cancer, pulmonary disease, and dermatological diseases [8–12]. Machine learning (ML) techniques have also shown their credibility during the COVID-19 pandemic and have aided medical professionals in identifying the coronavirus disease along with its levels [13].

In dermatology, effective diagnosis and prediction have been achieved by different machine learning methods. Scalp analysis systems have been developed utilizing SVM and KNN to classify scalp images. Scalp images have been used for classification of conditions such as dandruff with the employment of machine learning techniques of SVM, KNN, and decision trees [14–20]. All these techniques use scalp and/or skin images to develop prediction models. To the best of our knowledge, so far, none of the machine learning techniques has been applied on human hair images.

In this paper, we propose a framework that encapsulates practical application in effectively classifying alopecia areata and healthy hairs using hair images as previous work has been carried out with scalp and skin images only. Our proposal demonstrates the practical application of machine learning techniques for distinguishing alopecia areata. The results from our study exhibit the future potential of this framework to distinguish hair disorders that cannot be determined by the naked eye.

## 2. Related Works

Most researchers have used scalp images to extract skin features characteristic of alopecia areata. A trichoscopy method was proposed that involved extraction of hair loss feature by processing of scalp images using encapsulated techniques such as grid line selection and eigenvalue. The system was novel in terms of using a combination of computer vision and image processing techniques for alopecia areata diagnosis [14]. In another study, an automated classification method for the early diagnosis and treatment of alopecia was proposed using artificial neural networks (ANN). The system used a feedforward artificial neural network, and the results exhibited an accuracy of 91% [15]. In another work, scalp images were classified according to three scalp conditions namely, alopecia areata, dandruff, and normal hair. The classification yielded an accuracy of 85% [16]. In another study, texture analysis was executed on scalp images using Severity of Alopecia Tool (SALT) score. The proposed system permitted analysis of hair density changes exhibited in alopecia areata [17].

Other systems have used scalp images to analyze hair density and loss that manifest due to various reasons, including alopecia areata. A system referred to as TrichoScan was developed using epiluminescence microscopy to analyze hair cover in scalp images of people with androgenic alopecia (AGA). Four parameters, namely, hair density, hair diameter, hair growth rate, and anagen/telogen ratio, were extracted, and the results reported a correlation of approximately 91% [21]. In another system, hair loss was diagnosed via the application of artificial neural networks (ANNs). Scalp images were acquired from three hundred and forty-eight participants, and the results of the study exhibited that artificial neural networks can be utilized for detecting hair loss [22]. Shih [23] captured forty microscopic scalp images with a magnification factor of eighty-five to propose a hair counting algorithm involving features such as density, diameter, length, and hair oiliness level. The algorithm was observed to be more accurate than the traditional Hough-based one and was more reliable in counting hairs on an individual's scalp as compared to manual counting.

Researchers have also used scalp images to develop machine learning models for diagnosing different diseases. An intelligent scalp analysis system was proposed employing different machine learning methods such as SVM, linear discriminant analysis (LDA), KNN, and decision trees. Classification was carried out between two groups, namely, bacteria 1 that pertains to blisters or boils in the scalp and bacteria 2 that comprises of scalp skin exhibiting red spots. The highest accuracy of 80% was achieved with the application of SVM [18]. Another scalp analysis system used optical coherence tomography (OCT) and machine learning to identify fungal infection. A-line features comprising of attenuation coefficient values and B-scan features involving texture parameters such as energy, kurtosis, and skewness were extracted from the captured scalp images. Classification was carried out between nondandruff and dandruff scalps with the application of machine learning algorithms including decision tree, SVM, neural network, and extreme learning machine (ELM). The highest accuracy of 87.5% was acquired via SVM followed by neural network, decision tree, and ELM with 83.3%, 79.16%, and 75.23% accuracies, respectively [19]. A webcam and microscope camera sensor system was proposed for executing a hair and scalp analysis with reference to the Norwood-Hamilton scale. *K*-means clustering was applied, and the level of baldness was determined. The results exhibited accuracy in the range of 71% to 84% for different circumstances such as oily scalp, swollen/red scalp, and dry scalp [20].

The literature review demonstrates that no work has been done with hair images for identification of alopecia areata (hair disorder). Previous work has been carried out with dermoscopic and scalp images. Similar image preprocessing steps were used in [16]; however, the study made use of scalp images and applied only SVM with 85% accuracy. Furthermore, feature extraction techniques were also different in [16] as compared to our proposed framework. Hence, our work demonstrates a novel and innovative framework for classifying alopecia areata using color, texture, and shape as features and SVM and KNN as classification algorithms.

## 3. Materials and Methods

### 3.1. Datasets

#### 3.1.1. Healthy Hair Image Dataset

A total of 200 healthy hair images have been retrieved from the Figaro1k dataset. Figaro1k is a publicly available dataset containing different classes of hair images such as straight, wavy, and curly [24]. A normalization procedure has been applied on the dataset to ensure that the size and the aspect ratio of every image are the same [24].  Table 1 lists a few healthy hair images from the Figaro1k dataset that have been utilized in this study.

#### 3.1.2. Alopecia Areata Image Dataset

A total of 68 hair images of alopecia areata are retrieved from the Dermnet dataset. The dataset available on Dermnet comprises of twenty-three categories of dermatological diseases, including alopecia areata [25]. Another type of disease images includes that of eczema, seborrheic keratoses, tinea ringworm, bullous disease, poison ivy, and psoriasis [25].  Table 1 illustrates a few alopecia areata images that we utilized.

### 3.2. Proposed Framework with SVM and KNN

**Figure 1:**
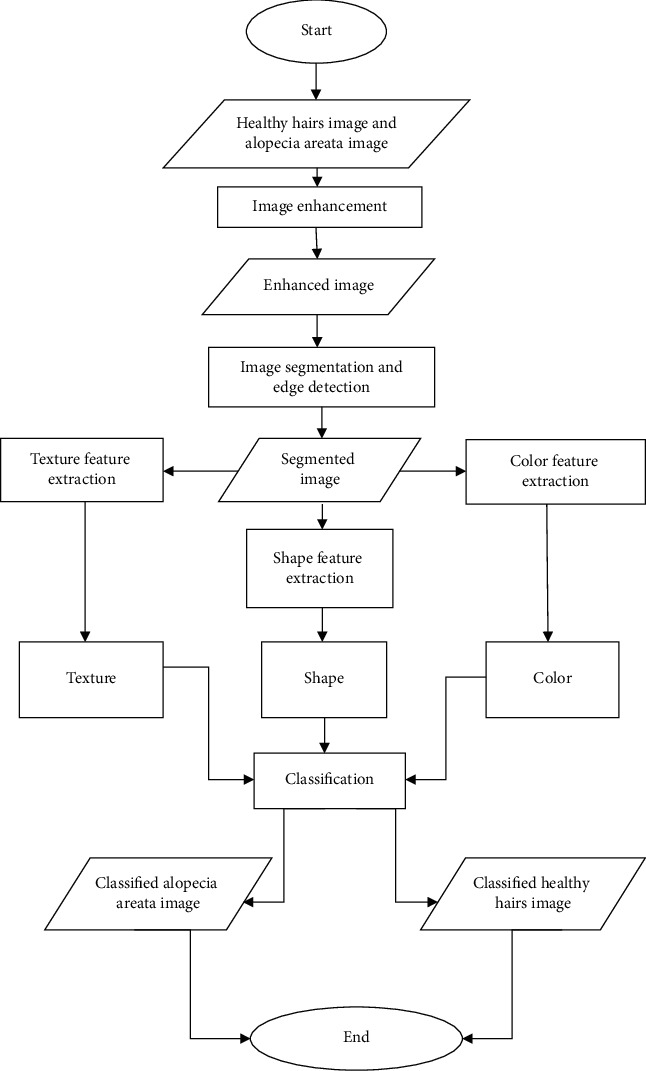
Proposed flow process of the classification framework.

To ensure that our data comprising the sample input images is organized and error-free, the dataframe function from Pandas Python Library is utilized to eliminate unwanted rows and columns and to clean the images. The code is written using Python on a Linux workstation utilizing the TensorFlow package with NVidia Titan GPU. The classification technique is executed with the aid of two machine learning methods, support vector machine (SVM) and *k*-nearest neighbor (KNN). The proposed flow process of the classification framework is exhibited in Figure 1. It starts initially with the input sample images of healthy hairs and alopecia areata. This is followed by image enhancement process that permits getting rid of any unwanted deformation in the images. Following image enhancement, image segmentation and edge detection are carried out. Furthermore, three features, namely, color, texture, and shape, are extracted, and the classification process is executed. Empirical studies represent that more reliable results can be acquired if 20-30% of data is used for testing and 70-80% for training [26]. Hence, in this study, 70% of the images have been used for model training and the remaining 30% are utilized for testing. The end result is the classification of an image into alopecia areata (class 0) or healthy hairs (class 1).

### 3.3. Image Preprocessing

#### 3.3.1. Image Enhancement

**Figure 2:**
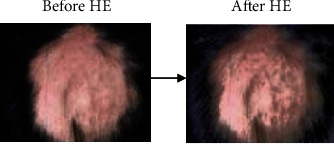
Sample outcomes of HE for alopecia areata.

**Figure 3:**
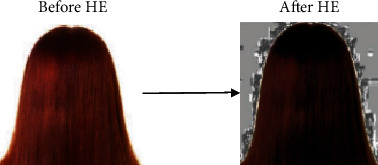
Sample outcomes of HE for healthy hair.

The technique of image enhancement relates to improving the contrast, brightness, and the pixel luminance values [27]. In this study, the sklearn.preprocessing library part of scikit-image processing that involves a vast array of techniques for image enhancement and image segmentation has been employed. The technique pertaining to histogram equalization is used to enhance the sample input images. Histogram equalization (HE) tends to improve areas of lower local contrast and enhances the intensities that ultimately lead to increase in the global contrast of the sample input images [28]. In our study, histogram equalization (HE) is executed by converting the RGB image into an equivalent hue-saturation-value (HSV) image format. Histogram equalized intensity matrix is produced, and the image is enhanced. This is exhibited by Figures 2 and 3 demonstrating the sample image of alopecia areata and healthy hair before and after histogram equalization, respectively.

#### 3.3.2. Image Segmentation and Edge Detection

**Figure 4:**
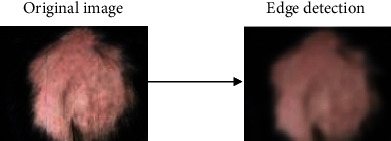
Sample outcomes of edge detection for alopecia areata.

**Figure 5:**
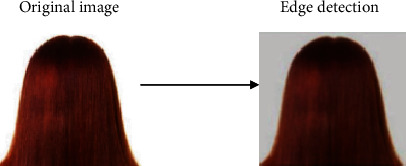
Sample outcomes of edge detection for healthy hair.

The image segmentation operation pertains to dividing the constituents of an image into desired fragments or sections that have similar features like texture, intensity, and pixel values [29]. In this study, image segmentation has been carried out via the resize operation and edge detection. The resize operation resizes an image by a given scaling factor or dimension. In this study, the resize dimension was set to 64; hence, a segmented output image with a dimension factor of 64 was generated. A major technique of image segmentation is edge detection. Edge detection is used to identify curves in an image that follow a path pertaining to rapid change in the intensity of the image [30]. In this study, the antialiasing technique in relation to edge detection and as part of the scikit-image processing library of Python has been utilized. The antialiasing operation is set to true to denote that the rough edges in the images are smoothened. Figures 4 and 5 show sample images of alopecia areata and healthy hair before and after edge detection, respectively.

### 3.4. Feature Extraction

Our study involves the extraction of three features of color, texture, and shape from each input sample image. The libraries of Python used for color, shape, and texture feature extraction include cv2 and skimage.

#### 3.4.1. Color Feature

In this study, the images have been converted to a NumPy array involving a list of color pixel values of RGB. The cv2 library is used to compute the mean of each of the three color channels including red, green, and blue. The first mean value that the cv2 library generates is of the blue channel, the second is of the green channel, and the third relates to the red channel. The cv2 library stores RGB images as NumPy array in a reverse order; hence, the first value corresponds to the blue channel, the second to the green channel, and the third value to the red channel.

#### 3.4.2. Texture Feature

As part of this study, the skimage library and the cv2 library of Python have been imported to exploit scikit's image processing capability. Local Binary Patterns (LBPs) are employed as texture descriptors to compute the local representation of the texture feature. The local representation that aids in extracting the texture feature is constructed by comparing each pixel of the image with its surrounding neighborhood of pixels. The texture is extracted using LBPs, where gc is the intensity value of the central pixel and gp is the intensity of the neighboring pixel with index *p* as specified in the following equation:

(1) $$\begin{array}{c} \text{LBP}\left(\text{gp}x,\text{gp}y\right)=\overset{p-1}{\underset{p=0}{\sum }}S\left(\text{gp}-\text{gc}\right)\times 2^{p}. \end{array}$$

The function *S* can be expressed as

(2) $$\begin{array}{c} S\left(x\right)=\left\{\begin{array}{c} 1\text{ if }x\geq 0\\ 0\text{ if }x<0 \end{array}\right\}. \end{array}$$

#### 3.4.3. Shape Feature

The OpenCV library of Python that has been imported in our study utilizes Hu moment shape descriptor to extract the shape feature, where *h* denotes the computed Hu moment and *η* represents the normalized central moment. The Hu moment shape descriptor is exhibited in Equation (3) and Equation (4). Central moment is involved in the computation of Hu moments as they shift the center of the image to the centroid region. Furthermore, Hu moments incorporating central moments tend to be invariant to translation, scale, and rotation that help in the extraction of the shape feature. Hu moments are able to extract shape features by quantifying the outline of the sample input images thus yielding the NumPy array form of the images. Finally, the flatten operation flattens the NumPy array to produce the shape feature vector.

(3) $$\begin{array}{c} h_{O}=\eta_{2O}+\eta_{O2}, \end{array}$$

(4) $$\begin{array}{c} h_{1}={\left(\eta_{2O}-\eta_{O2}\right)}^{2}+4\eta_{11}^{2}. \end{array}$$

### 3.5. Classification

**Figure 6:**
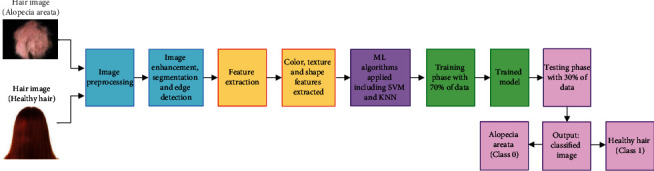
Architecture of the proposed framework. Top left: sample input images of alopecia areata and healthy hairs. Turquoise block: image preprocessing. Orange block: color, texture, and shape feature extraction. Purple block: SVM and KNN application. Green block: training phase. Pink block: testing phase.

In this study, support vector machine (SVM) and *k*-nearest neighbor (KNN) have been utilized for classifying healthy and alopecia areatahair images into their accurate classes.  Figure 6 depicts the architecture of our framework, including the training and testing phases. The initial steps in the framework are concerned with image preprocessing and feature extraction. This is followed by model training with machine learning algorithms and then execution of the testing phase.

### 3.6. Mathematical Operations of Classifiers

#### 3.6.1. Mathematical Operations of SVM

Support vector machine (SVM) determines the linear and nonlinear separability with the aid of a hyperplane [31]. Its kernel method transforms two-dimensional nonlinearly separable data into higher dimensions that yield the optimal hyperplane to separate the data [32]. The kernel trick employs the multiplication of a kernel function *k* with the dot product *x*_*i*_ · *x*_*j*_ as represented by

(5) $$\begin{array}{c} \underset{\propto }{\max}\overset{m}{\underset{i=1}{\sum }}\propto_{i}-\frac{1}{2}\overset{m}{\underset{i=1}{\sum }}\overset{m}{\underset{j=1}{\sum }}\propto_{i}\propto_{j}y_{i}y_{j}K\left(x_{i}\cdot x_{j}\right). \end{array}$$

In this study, the radial basis function (RBF) has been used as the kernel function. It is also referred to as the Gaussian kernel and contains a parameter *γ* as shown in the following equation:

(6) $$\begin{array}{c} K\left(x_{i}.x_{j}\right)=\exp\left(-\gamma \left\|x_{i}-\right.{\left.x_{j}\right\|}^{2}\right). \end{array}$$

#### 3.6.2. Mathematical Operations of KNN

The *k*-nearest neighbor algorithm pertains to finding the nearest neighbors. The process involves finding the nearest point that lies close to the input point in a given dataset [33]. In this study, the neighbors are specified as three which denotes that for every new input data, the three closest neighbors will be evaluated for classification. The algorithm initially analyzes the Euclidean distance that transforms data points into mathematical values.

The Euclidean distance formula in Equation (7) finds the distance between two points in a plane with coordinates (*x*, *y*) and (*a*, *b*).

(7) $$\begin{array}{c} \text{dist}\left(\left(x,y\right),\left(a,b\right)\right)=\sqrt{{\left(x-a\right)}^{2}+{\left(y-b\right)}^{2}}. \end{array}$$

### 3.7. SPSS Analysis

The paired sample *T*-test was performed via Statistical Package for Social Sciences (SPSS), IBM SPSS Statistics for Windows, Version 22.0. Armonk, NY: IBM Corp., on the accuracies generated from both SVM and KNN. The number of accuracy samples for both the algorithms was thirty.

## 4. Results and Evaluation

### 4.1. Performance Evaluation

**Figure 7:**
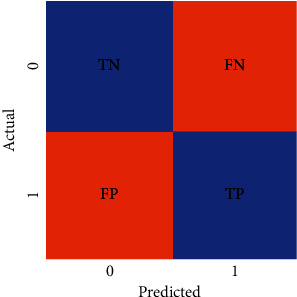
Confusion matrix.

The performance evaluation of support vector machine (SVM) and *k*-nearest neighbor (KNN) is evaluated using confusion matrices. The confusion matrix exhibited in Figure 7 demonstrates the predicted outcomes for the two classes. The two classes, alopecia areata and healthy hairs, have been denoted by 0 and 1, respectively. When the actual value is 1 and the predicted value is also 1, then the outcome is true positive (TP); otherwise, the outcome is false negative (FN). On the contrary, when the actual value is 0 and the predicted value is also 0, then the outcome is true negative (TN); otherwise, false positive (FP) is generated.

**Figure 8:**
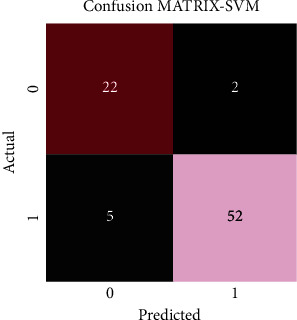
Confusion matrix of SVM illustrating the classified images of both alopecia areata (class 0) and healthy hairs (class 1).

Figure 8 exhibits the confusion matrix formulated after application of support vector machine (SVM) and represents that out of the 81 images tested, 74 images were classified accurately, thus yielding an accuracy of 91.4%. 22 images were classified as alopecia areata, and 52 images were classified as healthy hairs.

**Figure 9:**
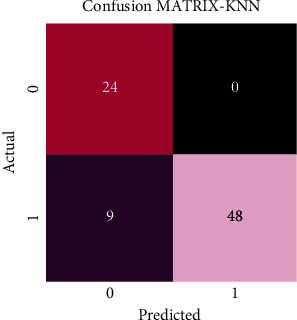
Confusion matrix of KNN illustrating the classified images of both alopecia areata (class 0) and healthy hairs (class 1).

Figure 9 exhibits the confusion matrix generated after the application of *k*-nearest neighbor (KNN) and shows that out of the total 81 images tested, 72 images were classified accurately, thus yielding an accuracy of 88.9%. 24 images were classified as alopecia areata, and 48 images were classified as healthy hairs. The reported accuracies were achieved after 10-fold cross-validation.

The performance and classification results for SVM and KNN are shown in Table 2. The accuracy for both SVM and KNN can be calculated by dividing the number of truly classified images by the total number of test images and multiplying the result with 100 as expressed in the following equation:

(8) $$\begin{array}{c} \text{Accuracy}=\frac{\text{TP}+\text{TN}}{\text{FP}+\text{FN}+\text{TP}+\text{TN}}\times 100\%. \end{array}$$

Equation (9) demonstrates the results obtained via SPSS analysis.

(9) $$\begin{array}{c} t\;\left(29\right)=4.744,\\ p<0.001. \end{array}$$

### 4.2. Performance Metrics

The sklearn library in Python helps to compute the true positives and false positives and true negatives and false negatives [34]. Classification techniques encapsulate classification metrics, namely, precision, recall, and F1 score. Precision relates to the ability of a classifier to be precise, i.e., its capacity to not to mark a positive circumstance that is actually negative. Recall is the ability of a classifier to identify all the true positives. Recall can be defined for each class as the ratio of true positives to the summation of true positives and false negatives [35]. F1 score can be defined as a score that exhibits the relationship between precision and recall [35]. In order to evaluate and analyze the effectiveness of the classification framework, the performance metrics shown in Equations (10), (11), and (12) including precision, recall, and F1 score were employed, where TP is true positive, TN is true negative, FP is false positive, and FN is false negative. The values of these performance metrics have been computed as per their formulas and are represented in Table 3.

(10) $$\begin{array}{c} \text{Precision}=\frac{\text{TP}}{\text{TP}+\text{FP}}\times 100\%, \end{array}$$

(11) $$\begin{array}{c} \text{Recall}=\frac{\text{TP}}{\text{TP}+\text{FN}}\times 100\%, \end{array}$$

(12) $$\begin{array}{c} \text{F}1\text{ score}=\frac{2*\text{precision}*\text{recall}}{\text{precision}+\text{recall}}\times 100\%. \end{array}$$

## 5. Discussion

### 5.1. Principal Findings

The objectives of this study were to extract the color, texture, and shape features from healthy and alopecia areata hair images and apply machine learning algorithms including support vector machine (SVM) and *k*-nearest neighbor (KNN) to execute classification of the images. SVM can classify linear and nonlinear data by generating a line or a hyperplane [31]. The RBF kernel method employed in our study aids to transform the data that yields the optimal hyperplane and in turn generates higher accuracy. On the contrary, KNN uses the Euclidean distance function to compute the probability of the test inputs that are closer to the data points [36]. Our study demonstrates better performance of SVM as compared to KNN when classifying hair images into healthy versus alopecia areata.

Other systems that have used the same machine learning techniques have also shown higher accuracies. For example, a skin lesion classification system based on support vector machine (SVM) and *k*-nearest neighbor (KNN) resulted in the accuracies of 89.50% and 82.00% for support vector machine (SVM) and *k*-nearest neighbor, respectively [37]. In another study, dermoscopic images were utilized for the classification of skin cancer using support vector machine (SVM), *k*-nearest neighbor (KNN), and random forest. The results demonstrated that support vector machine (SVM) performed better than the other two classifiers [38]. Better performance of support vector machine (SVM) lies in its mathematical operations. Hence, it can be deduced that the higher accuracy of 91.4% by support vector machine (SVM) is due to the use of kernel function that transforms the data into higher dimensions and yields the optimal hyperplane.

The strength of our proposed framework lies in being the first of its kind to classify alopecia areata and healthy hairs using hair images. The limitation of this study includes applying machine learning (ML) techniques on a limited dataset with no clinical data being collected. Nevertheless, it has been widely observed that deep learning techniques such as convolutional neural network (CNN) tend to generate high accuracies [39]. Furthermore, deep learning applications do not involve image preprocessing and feature extraction [39, 40]. An intelligent scalp analysis system was proposed employing convolutional neural network (CNN), and results exhibited accuracy of 89.77% [18]. Hence, future work can be carried out using CNN so that even better classification performance can be achieved.

## 6. Conclusion

This study proposed a classification framework for healthy hairs and alopecia areata using hair images with features including color, texture, and shape being extracted and support vector machine (SVM) and *k*-nearest neighbor (KNN) being applied. The application of support vector machine (SVM) and *k*-nearest neighbor (KNN) presented an accuracy of 91.4% and 88.9%, respectively. These accuracies exhibit that the proposed classification framework has been found to be successful and robust in classifying two different sets of hair images. However, future work with deep learning techniques such as convolutional neural networks (CNN) can also be carried out and integrated with the existing system.

## Figures and Tables

**Table 1:** Sample input images of alopecia areata and healthy hairs.

Classes	Images
Alopecia areata	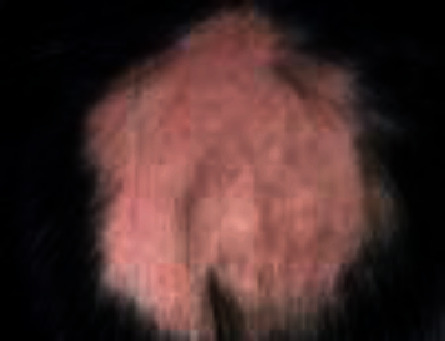 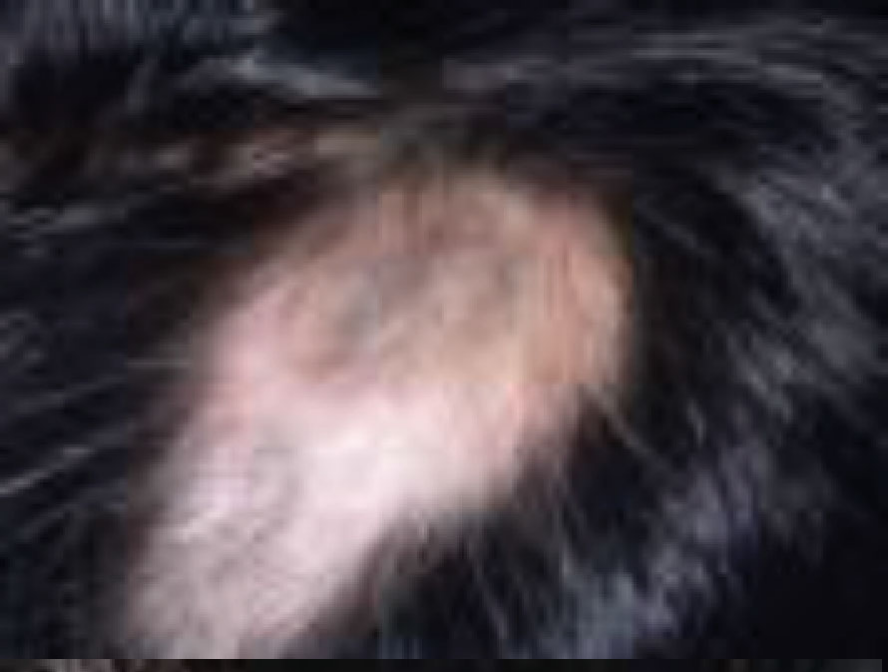 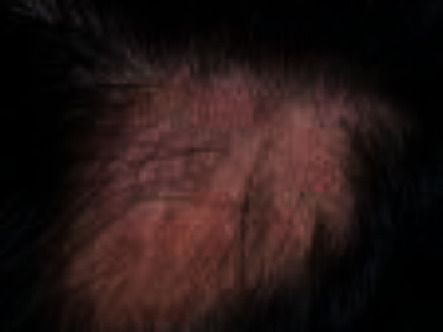

Healthy hair	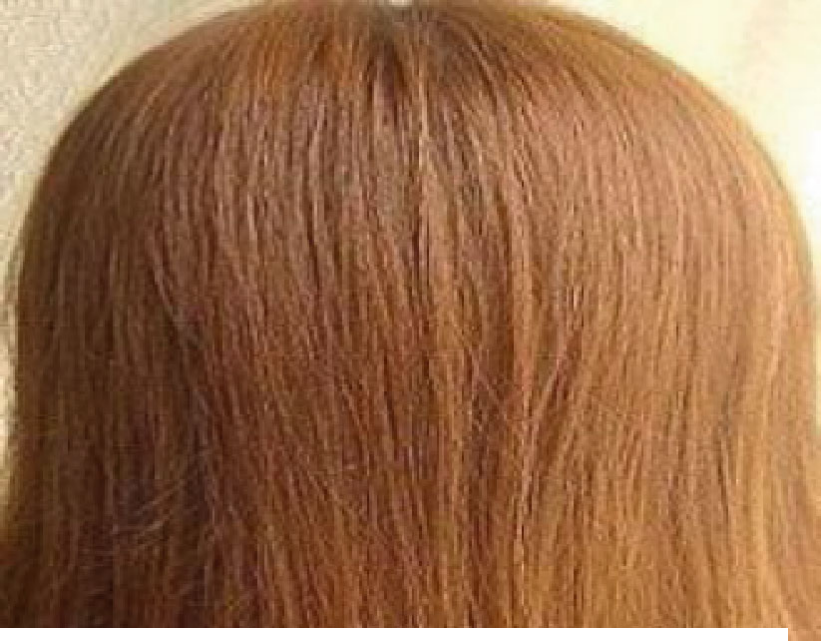 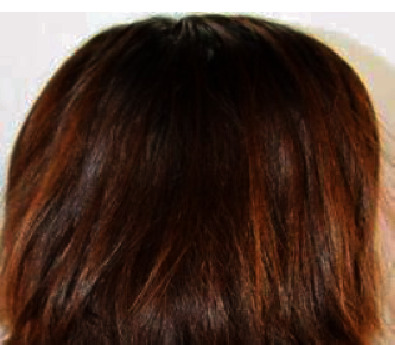 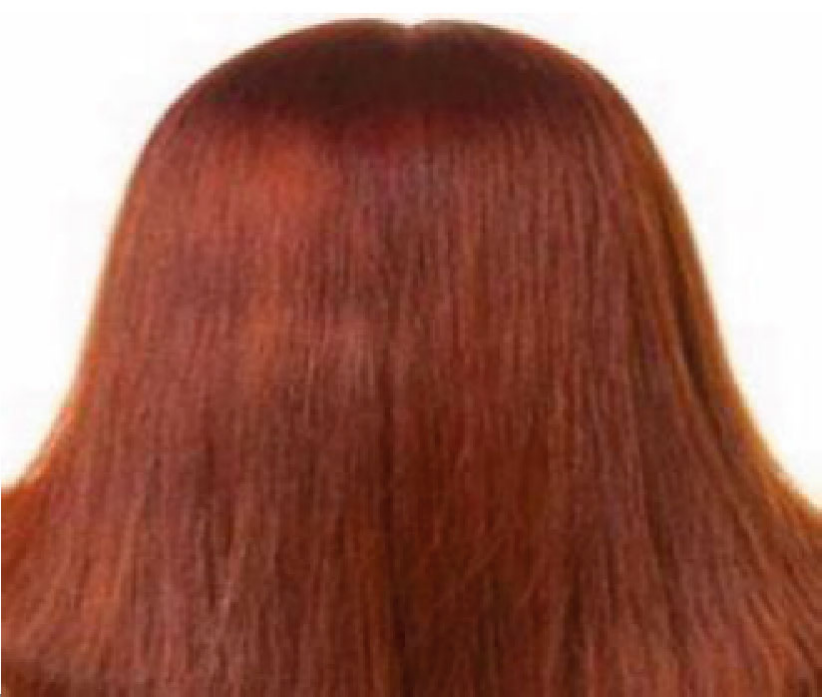

**Table 2:** Classification results of SVM and KNN.

ML algorithm	Test images	TRUE classification	Validation	% accuracy
SVM	81	74	10-fold	91.4
KNN	81	72	10-fold	88.9

**Table 3:** Values of performance metrics including precision, recall, and F1 score for both alopecia areata (class 0) and healthy hair (class 1).

ML algorithm	Class	Precision (%)	Recall (%)	F1 score (%)
SVM	0	81.5	91.7	86.3
SVM	1	96.3	91.2	93.7
KNN	0	72.7	100	84.2
KNN	1	100	84.2	91.4

## Data Availability

The datasets used are available at the following links: (1) http://projects.i-ctm.eu/it/progetto/figaro-1k and (2) https://www.kaggle.com/shubhamgoel27/dermnet.
